# Pirfenidone alleviates pulmonary fibrosis in vitro and in vivo through regulating Wnt/GSK-3β/β-catenin and TGF-β1/Smad2/3 signaling pathways

**DOI:** 10.1186/s10020-020-00173-3

**Published:** 2020-05-24

**Authors:** Qun Lv, Jianjun Wang, Changqing Xu, Xuqing Huang, Zhaoyang Ruan, Yifan Dai

**Affiliations:** Department of Pneumology, The Affiliated Hospital of Hangzhou Normal University, No. 126, Wenzhou Road, Hangzhou, 31000 Zhejiang China

**Keywords:** Pirfenidone, Pulmonary fibrosis, Bleomycin, TGF-β1, Signaling pathway

## Abstract

**Background:**

Pirfenidone (PFD) is effective for pulmonary fibrosis (PF), but its action mechanism has not been fully explained. This study explored the signaling pathways involved in anti-fibrosis role of PFD, thus laying a foundation for clinical application.

**Methods:**

Pulmonary fibrosis mice models were constructed by bleomycin (BLM), and TGF-β1 was used to treat human fetal lung fibroblasts (HLFs). Then, PFD was added into treated mice and cells alone or in combination with β-catenin vector. The pathological changes, inflammatory factors levels, and Collagen I levels in mice lung tissues were assessed, as well as the activity of HLFs was measured. Levels of indices related to extracellular matrix, epithelial-mesenchymal transition (EMT), Wnt/GSK-3β/β-catenin and TGF-β1/Smad2/3 signaling pathways were determined in tissues or cells.

**Results:**

After treatment with BLM, the inflammatory reaction and extracellular matrix deposition in mice lung tissues were serious, which were alleviated by PFD and aggravated by the addition of β-catenin. In HLFs, PFD reduced the activity of HLFs induced by TGF-β1, inhibited levels of vimentin and N-cadherin and promoted levels of E-cadherin, whereas β-catenin produced the opposite effects to PFD. In both tissues and cells, Wnt/GSK-3β/β-catenin and TGF-β1/Smad2/3 signaling pathways were activated, which could be suppressed by PFD.

**Conclusions:**

PFD alleviated pulmonary fibrosis in vitro and in vivo through regulating Wnt/GSK-3β/β-catenin and TGF-β1/Smad2/3 signaling pathways, which might further improve the action mechanism of anti-fibrosis effect of PFD.

## Background

Pulmonary fibrosis (PF) is a diffuse pulmonary inflammatory disease, which mainly involves pulmonary interstitium, alveolar epithelial cells and pulmonary blood vessels (Meyer 2017). The disease has many causes, including related diseases (such as rheumatoid arthritis and lupus erythematosus), environmental factors (such as particulate matter and smoking), and the adverse effects of some drugs (such as bleomycin (BLM)) (Noble et al. 2012). In the pathological changes, the disease was mainly manifested by proliferation of lung stromal cells, excessive deposition of extracellular matrix and inflammatory response, which will lead to the impediment of eliminating apoptosis or damaged cells, thus stimulating neighboring cells and inducing dysregulation of transforming growth factor beta (TGF-β) (Tomos et al. 2017). In progress, idiopathic PF (IPF) is a clinically common and representative chronic fibrotic lung disease with unknown etiology, characterized by progressive pulmonary fibrosis, high disability rate and mortality, and a median survival time of only 3–5 years (Richeldi et al. 2017). Therefore, exploring new drugs for treating PF and verifying its mechanism have become a challenge for clinical workers.

Pirfenidone (PFD) is a pleiotropic pyridine compound with the effect of improving fibrosis, inflammatory response and oxidative stress response (Lopez-de la Mora et al. 2015). In the early stage of research, PFD was used in the treatment of hermansky-pudlak syndrome (HPS)-associated pulmonary fibrosis, which initially showed that the drug may delay the decline of forced vital capacity (FVC) (Gahl et al. 2002). In subsequent in vitro and in vivo experiments (Stahnke et al. 2017; Komiya et al. 2017; Medina et al. 2019), PFD has been defined to inhibit the production and release of pro-fibrotic and pro-inflammatory cytokines such as TGF-β, tumor necrosis factor-alpha (TNF-α) and interleukin (IL)-6, thereby postponing fibroblast proliferation and collagen deposition. PFD intervention reduced the level of TNF-α, and IL-6 in lung tissues, inhibited the epithelial-mesenchymal transition and pulmonary fibrosis in rat silicosis model, which effects may be related to the TGF-β1/smad pathway (Guo et al. 2019). PFD suppressed fibrotic fibroblast-mediated fibrotic processes via inverse regulation of lung fibroblast activity (Jin et al. 2019). In clinical application, PFD is the only clinical drug currently approved for the treatment of IPF (Kim and Keating 2015), and it is reported to be capable of restraining the fibrotic progression in diverse organs, including liver, heart, kidney, small intestine, skin and so on (Komiya et al. 2017; Meier et al. 2016; Li et al. 2017a; Li et al. 2017b). Nonetheless, although the therapeutic role of PFD in fibrosis-related diseases has been recognized, its mechanism of action in vivo and in vitro is still not fully understood. Therefore, exploration of the action mechanism and latent signaling pathways of PFD in PF, especially TGF-β, TNF-α and IL-6, contributes to better understanding on the role of drugs, thus laying a foundation for clinical application.

Herein, BLM, a widely used drug in animals, was used to to induce pulmonary fibrosis of animals, and TGF-β1 was used to treat human fetal lung fibroblasts (HLFs) to induce phenotypic transformation. Then, in vitro and in vivo models were processed using PFD or β-catenin, and changes in signaling pathway-related indicators in mice lung tissue and HLFs after treatment were further investigated. The purpose of this study was to explore the therapeutic mechanism of PFD for pulmonary fibrosis, so as to further supplement the drug mechanism.

## Methods

### Ethics statement

Animal experiments implemented in this study were approved by the Ethic Committee of the Affiliated Hospital of Hangzhou Normal University, and followed the guidelines of Animal Care and Institutional Ethical in China.

### Modeling administration

A total of 30 C57BL/6 mice (7 ~ 8 weeks old, male, weight 18 ~ 22 g) were purchased from Vital River Laboratories (Beijing, China). All mice were fed in controlled bioclean conditions (temperature of 20 ~ 25 °C, relative humidity of 45 ~ 60%, in a 12:12 h light/dark cycle), and allowed to acquire water and food freely before following experiments. Mice were randomly allotted into 5 groups on average: control, model, PFD, PFD + NC, and PFD + β-catenin groups.

Mice were collected after feeding for 1 week, and then anesthetized by intraperitoneal injection of 10% chloral hydrate (0.3 mL/100 g). Next, mice in the control and model groups respectively received intratracheal injection of normal saline or BLM (5 mg/kg, Nippon Kayaku, Tokyo, Japan; drug import registration number: H20090885; batch number: 640412), and followed by oral normal saline from day 2 to 21. Mice in PFD group received oral PFD (300 mg/kg, Tianjin Jin Yao Pharmaceutical, Co., Ltd., Tianjin, China; Lot: 1510002; CAS: 53179–13-8) from day 2 to 21 after 5 mg/kg BLM injection, and those who received intraperitoneally injection of empty viral vector or equal β-catenin adenovirus vector (constructed by Vertor Builder, Guangzhou, China) into lung were severally regarded as PFD + NC group and PFD + β-catenin group. On day 21, the mice were sacrificed by overdose anesthesia, and ambilateral lungs were rapidly separated for subsequent experiments. The subsequent studies with the rats were done with blinded analysis.

### Pathological identification

Lung tissues were routinely fixed in 4% formaldehyde, embedded in paraffin and then sliced into sections (approximately 5 μm thick). Hematoxylin-eosin (HE) and Masson trichrome stainings (Solarbio, Beijing, China) were performed to the samples according to the manufacturer’s instructions. The pathological changes and collagen deposition in lung tissues were observed and photographed with a light microscope (EVOS FL Auto Cell Imaging System, USA).

### Enzyme-linked immunosorbent assay (ELISA) assay

The lung tissues were centrifuged (2500 x*g*) for 10 min at 4 °C, and collected the supernatant for the analysis of TNF-α (#SEKM-0034, Solarbio, Beijing, China) and IL-6 (#SEKM-0007, Solarbio, Beijing, China) levels by corresponding ELISA assay kits. All procedures were implemented following the manufacturers’ instructions. And the results of these two indices in lung tissue supernatant were normalized with the protein concentration measured by Coomassie blue staining kit (Beyotime Biotechnology Co., Ltd. Shanghai, China), and the levels were indicated as ng or pg per protein (mg).

### Immunohistochemistry

Immunohistochemistry was carried out to authenticate the content of Collagen I (Col-I) in lung tissues. In brief, the lung samples were stayed in 3% H_2_O_2_ for 30 min to inactivate endogenous peroxidase, and then blocked with 3% bovine serum albumin (BSA) for 30 min. Subsequently, the samples were hatched with primary antibody against anti-collagen I (ab6308, abcam, USA) overnight at 4 °C. A horseradish peroxidase (HRP)-labeled goat anti-mouse secondary antibody (ab205719, abcam, USA) was then added for another 1 h at room temperature. The samples were visualized by routine dehydration, transparent, diaminobenzidine (DAB) staining and neutral resin sealing. The images were collected under an inverted microscope (Eclipse TS-100, Nikon, Tokyo, Japan).

### Cell experiment

The HLFs were provided by the Cell Bank of the Chinese Academy of Sciences (Shanghai, China), and maintained in Ham’s F-12 K medium (Gibco, Carlsbad, CA) containing 10% fetal bovine serum (FBS, Gibco) in an incubator with 5% CO_2_ at 37 °C. After starvation for 24 h, cells were harvested and seeded in 96-well plates (5 × 10^3^ cells/well). Cells were transfected with empty viral vector or equal β-catenin adenovirus vector, and using un-transfected cells as negative control (NC). In addition, cultured HLFs were divided into control (cultured in medium), TGF-β1 (first incubated with TGF-β1 (5 ng/mL, #100–21, PeproTech, USA) for 24 h and then cultured in medium for another 48 h), PFD + TGF-β1 (first incubated with TGF-β1 for 24 h and then cultured in PFD (200 μg/mL) for another 48 h), PFD + TGF-β1 + NC (incubated with TGF-β1 for 24 h and in PFD for additional 48 h, followed by transfecting with empty viral vector), and PFD + TGF-β1 + β-catenin (incubated with TGF-β1 for 24 h and in PFD for additional 48 h, followed by transfecting withβ-catenin adenovirus vector) groups.

### Cell viability

The methylthiazolyldiphenyl-tetrazolium bromide (MTT) assay was performed to determine the viability of HLFs after treatment for 24 and 48 h. Briefly, 10 μL of MTT solution (Sigma-Aldrich, USA) was added to cells at the appropriate point for 4 h at 37 °C, and the optical density (OD) was read at a wavelength of 490 nm using the ELX-800 Biotek plate reader (Winooski, USA).

### Quantitative real-time polymerase chain reaction (qRT-PCR) assay

The mRNA expression levels of Col-I, Col-III, α-SMA, fibronectin and epithelial-mesenchymal transition (EMT)-related proteins (Vimentin, E-cadherin, N-cadherin) in mice lung tissues or HLFs were measured by qRT-PCR assay. For the detection, the total RNA of cells or tissues was isolated using the Trizol reagents (Invitrogen, Carlsbad, California, USA). NanoDrop Spectrophotometer (Thermo Fisher Scientific, Massachusetts, USA) and 1% agarose modified gel electrophoresis were respectively implemented to measure the concentration and completeness of isolated RNA. RNA (1 μg) was reverse transcribed into cDNA by the PrimeScript RT Master Mix Perfect Real Time (TaKaRa, Shiga, Japan) in line with the manufacturer’s instructions. The Applied Biosystems 7500 Real-Time PCR System (Applied Biosystems, Foster City, CA) was performed for 40 cycles of 95 °C for 30 min, 60 °C for 60 s, and 72 °C for 30 s. The sequences of primers were shown in Table 1 and synthesized by Gene Pharma (Shanghai, China). 2^-ΔΔCt^ method (Rao et al. 2013) was used to calculate mRNA expression, and GAPDH was served as the internal control.

**Table 1:** Primer base sequence

Gene	Forward (5′-3′)	Reverse (5′-3′)
**Col-I**	**CAATGGCACGGCTGTGTGCG**	**CACTCGCCCTCCCGTCTTTGG**
Col-III	TGGTCCCCAAGGTGTCAAAG	GGGGGTCCTGGGTTACCATTA
α-SMA	GGCAACCTCAAGAAGTCCC	GTGCAGCCATCCACAAGC
fibronectin	TGGAACTTCTACCAGTGCGAC	TGTCTTCCCATCATCGTAACAC
Vimentin	GACGCCATCAACACCGAGTT	CTTTGTCGTTGGTTAGCTGGT
E-cadherin	CGAGAGCTACACGTTCACGG	GGGTGTCGAGGGAAAAATAGG
N-cadherin	CTCCTATGAGTGGAACAGGAACG	TTGGATCAATGTCATAATCAAGTGCTGTA
GAPDH	**CACTGGGCTACACTGAGCAC**	**AGTGGTCGTTGAGGGCAAT**

### Western blot (WB) analysis

The protein levels of experimental proteins in mice lung tissues and HLFs were determined by WB assays. The total protein of cells and tissues were extracted by RIPA buffer (Solarbio, Beijing, China), and Bicinchoninic Protein Assay kit (BCA, Pierce, Rockford, IL, USA) was performed to detect the protein concentration. Fifty microgram of the total protein was exposed to 10% sodium dodecyl sulfate-polyacrylamide gel electrophoresis (SDS-PAGE, Beyotime, Shanghai, China) and then transferred onto polyvinylidene fluoride (PVDF) membranes. Next, the membranes were sealed with 5% fat-free milk for 2 h, and co-incubated with the primary antibodies at 4 °C overnight, including Col-I (1:1000, ab6308, Abcam, USA), Col-III (1:500, ab7778, Abcam, USA), α-SMA (1:500, ab5694, Abcam, USA), fibronectin (1:1000, ab2413), p-GSK-3β (Ser9, S9) (1:1000; #5558, Cell Signaling Technology, USA), β-catenin (1:1000; #8480, Cell Signaling Technology, USA), TGF-β1 (1:1000; ab92486, Abcam, USA), TGF-βRΙ (1:1000; ab31013, Abcam, USA), TGF-βRΙΙ (1:1000; ab186838, Abcam, USA), p-Smad2 Ser255 (1:1000; ab188334, Abcam, USA), p-Smad3 Ser213 (1:1000; ab63403, Abcam, USA), Total Smad2/3 (1:1000; #8685, Cell Signaling Technology, USA), Vimentin (1:1000; ab92547, Abcam, USA), N-Cadherin (1:1000; ab18203, Abcam, USA), E-Cadherin (1:1000; ab40772, Abcam, USA), and GAPDH (1:1000; ab181602 Abcam, USA) was taken as the internal reference. The corresponding secondary antibodies (goat anti-rabbit IgG H&L (HRP), ab6721, 1:7000; rabbit anti-mouse IgG H&L (HRP), ab3728, 1:7000; Abcam, USA) were added to the samples and incubated for 1 h at room temperature. The blots signals were developed by an enhanced chemiluminescence-detecting kit (Thermo Fisher, MA, USA).

### Statistical analysis

GraphPad Prism 8.0 software (GraphPad Software, Inc., La Jolla, CA, USA) was used for data analysis. The measurement data were presented as mean ± standard deviation (SD). The comparison between groups was performed by Student’s t-test or one-way analysis of variance (ANOVA). All experiments were repeated in triplicate. *P* < 0.05 was considered as statistically significant.

## Results

### Pathological changes in mice lung tissues

**Fig. 1:**
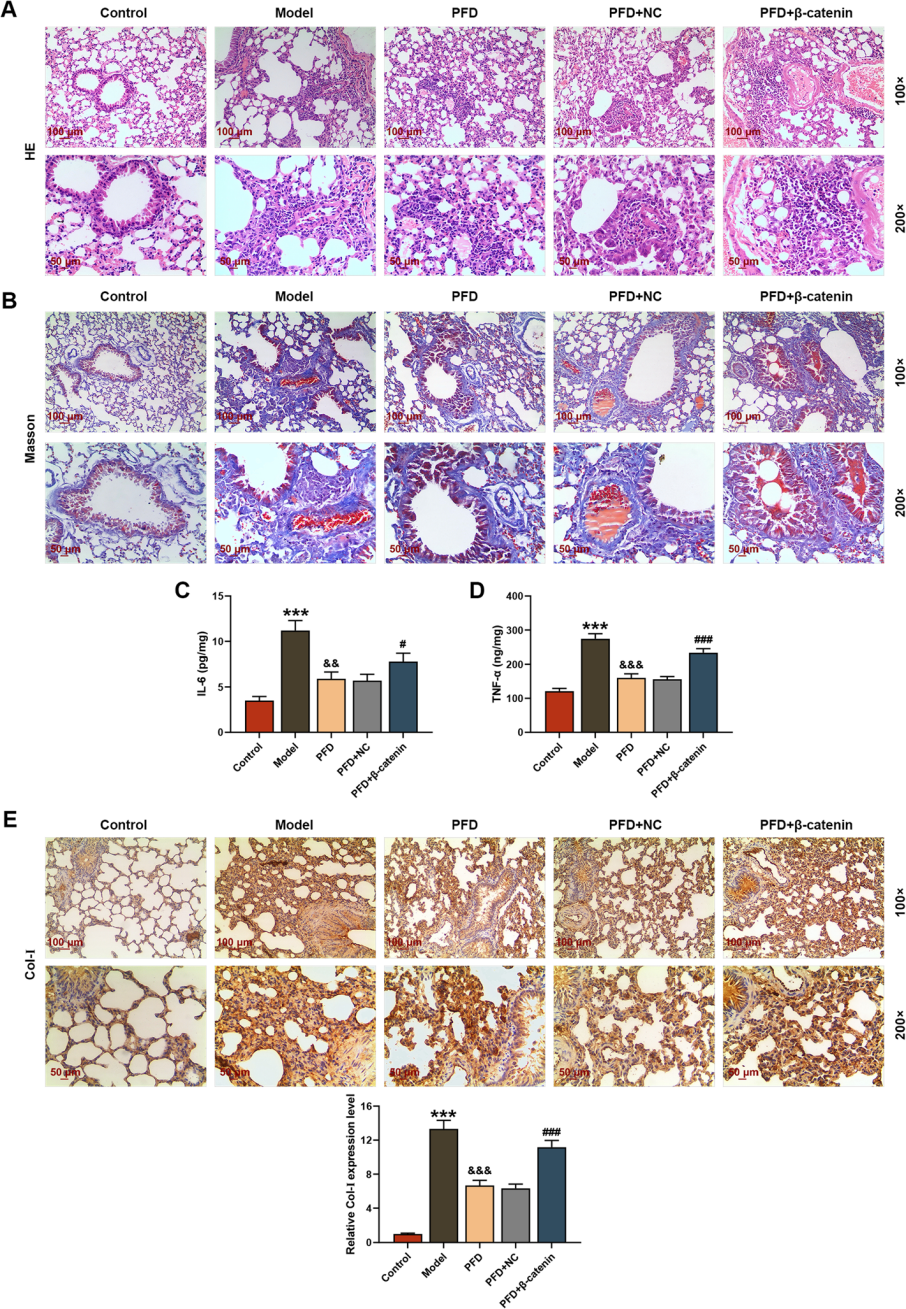
Pathological changes in mice lung tissues after treatment. In this figure, mice (*n* = 30) were randomly divided into equal 5 groups: control, model (Bleomycin (BLM)), pirfenidone (PFD), PFD + negative control (NC), and PFD + β-catenin. **a** Hematoxylin-eosin (HE) staining observed the structure and inflammatory reaction in in mice lung tissues after treatment. **b** Masson trichrome staining was performed to view the collagen deposition in lung tissues after treatment. **c**, **d** The levels of inflammatory factors IL-6 and TNF-α in lung tissue supernatant were measured by enzyme-linked immunosorbent assay (ELISA) assay. **e** The content of collagen I (Col-I) in lung tissues were detected through immunohistochemistry. ^***^P < 0.001, vs. control; ^&&^*P* < 0.01, ^&&&^*P* < 0.001, vs. model; ^#^*P* < 0.05, ^###^*P* < 0.001, vs. PFD + NC. *N* = 3

After treatment, the lung tissues of the mice were examined by pathological staining. Alveolar septa are thin layers of connective tissues between adjacent alveoli. When the elastic fibers in alveolar septa degenerate and alveolar septa becomes thickening, the retraction of the alveolar becomes poor, alveolar expands, and finally the respiratory function of the lungs decreases (Guo et al. 2019). According to the results of HE staining (Fig. 1a), the lung structure of mice in control group was relatively ideal, with thin alveolar septa and no inflammatory response, while the mice lung tissues in model group had severe inflammatory response, with significant thickening of alveolar septa, and infiltration of inflammatory cells. After the intervention of PFD, inflammatory cell infiltration and alveolar structure of mice lung tissues were improved in the PFD and PFD + NC group, while those in PFD + β-catenin group were aggravated due to the addition ofβ-catenin. As shown in Fig. 1b, Masson staining showed that the lung tissues were normal without obvious staining in the control group, whereas they showed large areas of blue staining, and a large amount of collagen fibers deposition in extracellular matrix in model group. However, extracellular matrix deposition in lung tissues of the PFD and PFD + NC groups was alleviated by the treatment of PFD, which that in PFD + β-catenin group was exasperated by the addition of β-catenin.

### PFD alleviated the PF induced by BLM

Through the detection of ELISA assays, we found that the levels of inflammatory factors TNF-α and IL-6 in the supersolution of mice lung tissues in model group were obviously increased, which were notably suppressed after treatment with PFD; whereas over-expression of β-catenin reversed the inhibiting effect of PFD on inflammatory factor release and thus elevating the levels of TNF-α and IL-6 in lung tissue supersolution (*P* < 0.05, Fig. 1c and d). In the collagen deposition, it could be seen from the immunohistochemical identification experiments that the Col-I deposition was relatively obvious in the model group, and the addition of PFD alleviated Col-I deposition in the lung tissues induced by BLM, while β-catenin rescued the effect of the PFD and promoted Col-I deposition (Fig. 1e).

### PFD played an anti-PF role by regulating Wnt/GSK-3β/β-catenin and TGF-β1/Smad2/3 signaling pathways

**Fig. 2:**
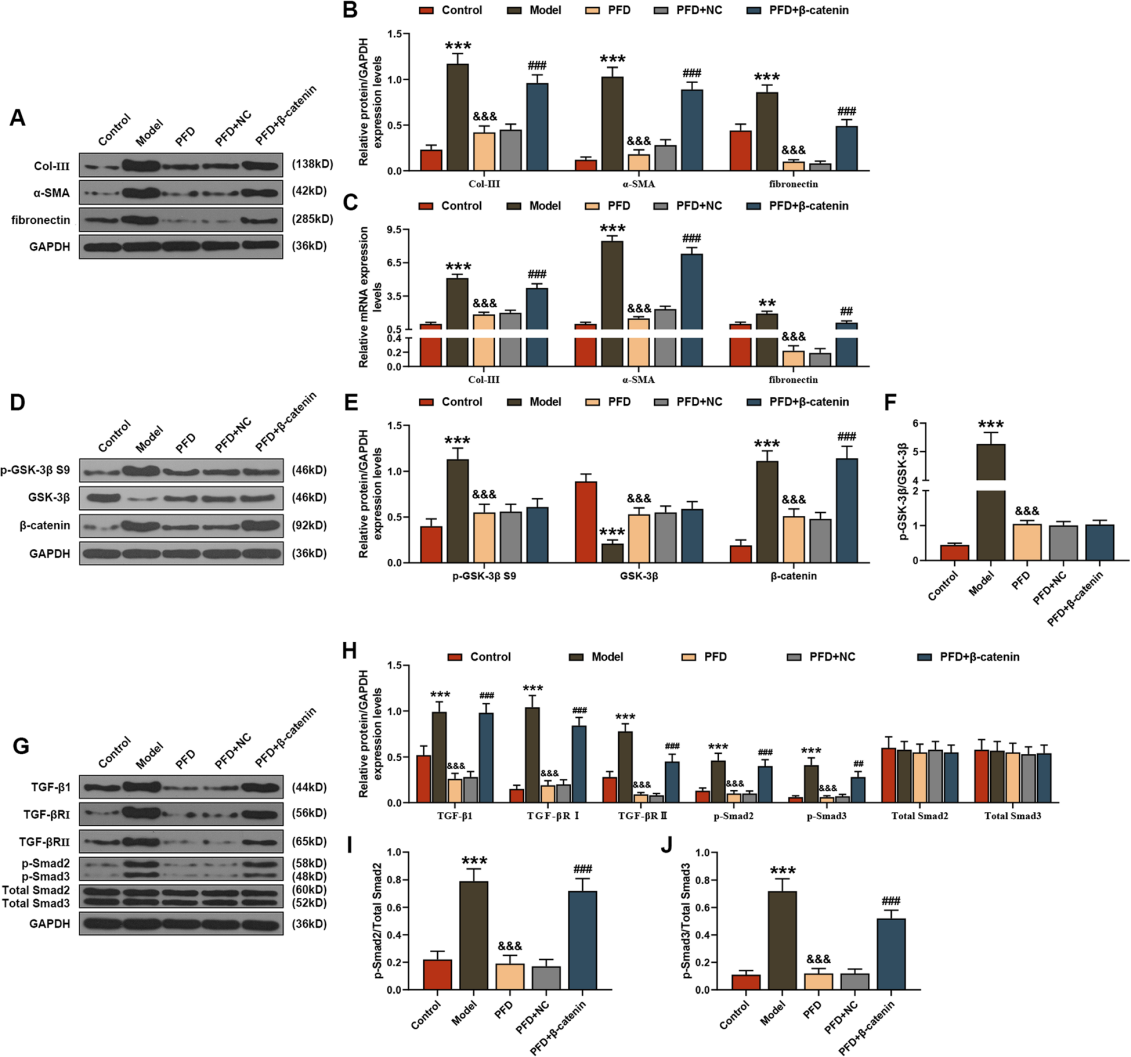
Pirfenidone (PFD) played an anti-pulmonary fibrosis role by regulating Wnt/GSK-3β/β-catenin and TGF-β1/Smad2/3 signaling pathways. In this figure, mice (*n* = 30) were randomly divided into equal 5 groups: control, model (Bleomycin (BLM)), PFD, PFD + negative control (NC), and PFD + β-catenin. The mRNA and protein levels of collagen (Col)-III, α-SMA and fibronectin in lung tissues were determined by (**a**, **b**) Western blot (WB) and (**c**) quantitative real-time polymerase chain reaction (qRT-PCR) assays. The protein levels of factors associated with (**d**-**f**) Wnt/GSK-3β/β-catenin (p-GSK-3β S9, β-catenin) and (**g**-**j**) TGF-β1/Smad2/3 (TGF-β1, TGF-βRΙ, TGF-βRΙΙ, p-Smad2/3) signaling pathways were measured by WB assay. ^**^P < 0.01, ^***^P < 0.001, vs. control; ^&&&^*P* < 0.001, vs. model; ^##^*P* < 0.01, ^###^*P* < 0.001, vs. PFD + NC. *N* = 3

In relevant mechanism, both qRT-PCR and WB analysis showed that the mRNA and protein levels of Col-III, α-SMA and fibronectin were significantly increased in the mice lung tissues of model group, but were decreased under the action of PFD, whereas the addition of β-catenin promoted the mRNA and protein levels of Col-III, α-SMA and fibronectin (*P* < 0.01, Fig. 2a-c). As for the potential signaling pathway, WB analysis revealed that markedly increasing trends were observed in all proteins related to Wnt/GSK-3β/β-catenin (p-GSK-3β S9, β-catenin) and TGF-β1/Smad2/3 (TGF-β1, TGF-βRΙ, TGF-βRΙΙ, p-Smad2/3) signaling pathways in lung tissues of model group, which could be mitigated by PFD; moreover, the effects of PFD on related proteins could be relieved by β-catenin (*P* < 0.01, Fig. 2d-j).

### PFD alleviated the damage of TGF-β1 to HLFs

**Fig. 3:**
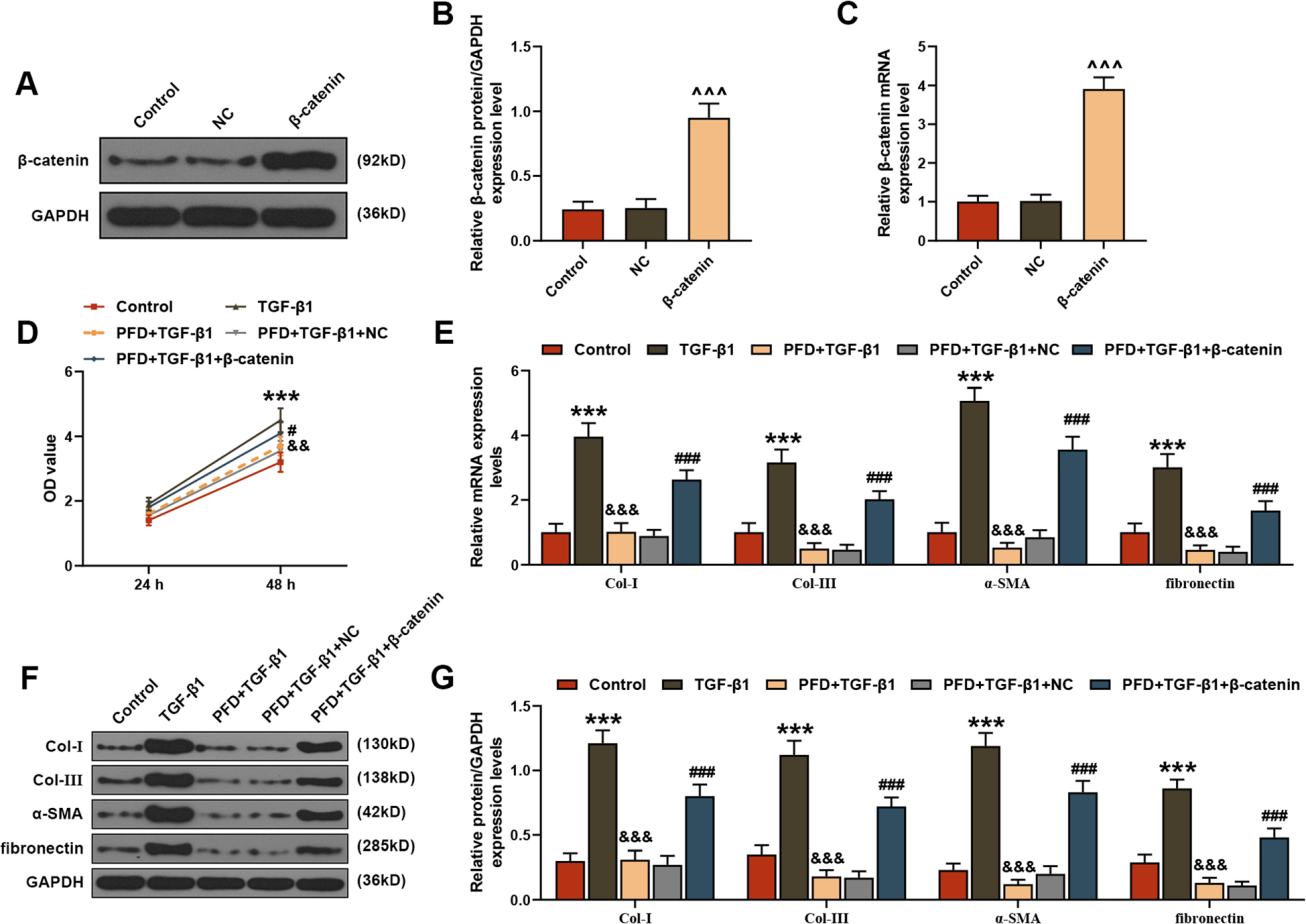
Pirfenidone (PFD) alleviated the damage of TGF-β1 to human fetal lung fibroblasts (HLFs). **a**, **b** Western blot (WB) and (**c**) quantitative real-time polymerase chain reaction (qRT-PCR) assays were used to measure the mRNA expression levels of β-catenin in HLFs after transfection with negative control (NC) or β-catenin vevtor, and untreated cells acted as controls. In following diagrams, HLFs were divided into control, TGF-β1, PFD + TGF-β1, PFD + TGF-β1 + NC and PFD + TGF-β1 + β-catenin groups. **d** The methylthiazolyldiphenyl-tetrazolium bromide (MTT) assay was performed to determine the viability of HLFs after treatment for 24 and 48 h. The mRNA and protein levels of collagen (Col)-I, Col-III, α-SMA and fibronectin in HLFs were determined by (**e**) qRT-PCR and (**f**, **g**) WB assays. ^^^^^*P* < 0.001, vs. NC; ^***^P < 0.001, vs. control; ^&&&^*P* < 0.001, vs. TGF-β1; ^###^*P* < 0.001, vs. PFD + TGF-β1 + NC. *N* = 3

**Fig. 4:**
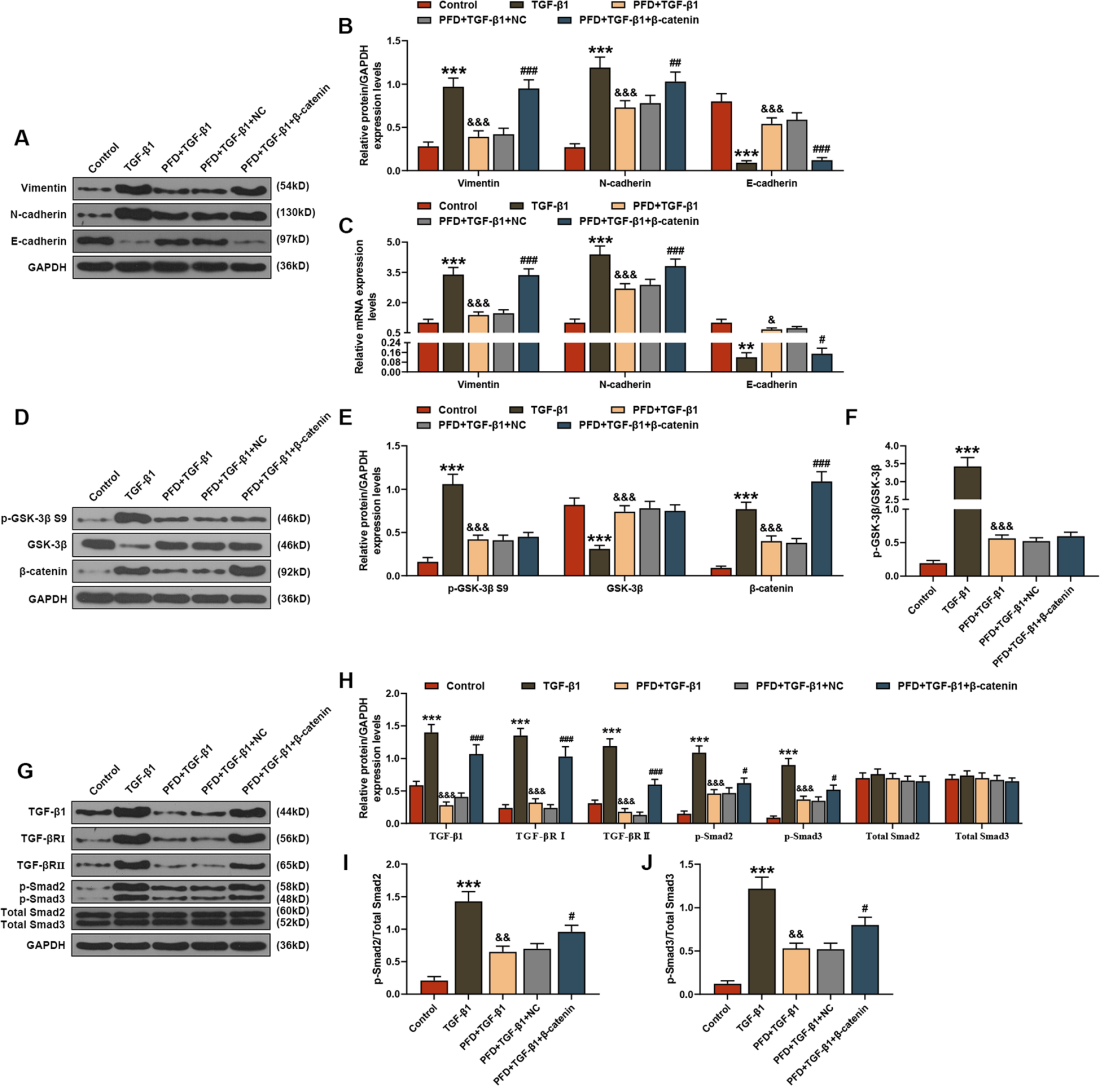
Pirfenidone (PFD) protected human fetal lung fibroblasts (HLFs) from TGF-β1 damage via Wnt/GSK-3β/β-catenin and TGF-β1/Smad2/3 signaling pathways. In this figure, HLFs were divided into control, TGF-β1, PFD + TGF-β1, PFD + TGF-β1 + NC and PFD + TGF-β1 + β-catenin groups. The mRNA and protein levels of epithelial-mesenchymal transition (EMT)-related proteins (Vimentin, E-cadherin, N-cadherin) in HLFs were determined by (**a**, **b**) Western Blot (WB) and (**c**) quantitative real-time polymerase chain reaction (qRT-PCR) assays. The protein levels of factors related to (**d**-**f**) Wnt/GSK-3β/β-catenin (p-GSK-3β S9, β-catenin) and (**g**-**j**) TGF-β1/Smad2/3 (TGF-β1, TGF-βRΙ, TGF-βRΙΙ, p-Smad2/3) signaling pathways were measured by WB assay. ^**^P < 0.01, ^***^P < 0.001, vs. control; ^&^*P* < 0.05, ^&&^*P* < 0.01, ^&&&^*P* < 0.001, vs. TGF-β1; ^#^*P* < 0.05, ^##^*P* < 0.01, ^###^*P* < 0.001, vs. PFD + TGF-β1 + NC. *N* = 3

In cell experiments, successful upregulation of β-catenin in HLFs could be found through detection of both qRT-PCR and WB analysis (*P* < 0.001, Fig. 3a-c). According to the MTT analysis, after treatment of 48 h, PFD effectively reduced the activity of HLFs induced by TGF-β1, which could be promoted by β-catenin (*P* < 0.05, Fig. 3d). Furthermore, this study also found that the mRNA and protein levels of Col-I, Col-III, α-SMA and fibronectin in TGF-β1-treared HLFs were obviously increased, which could be alleviated by PFD and deteriorated under the action of β-catenin (*P* < 0.001, Fig. 3e-g). Among the EMT-related indices, both qRT-PCR and WB analysis uncovered that TGF-β1 promoted the mRNA and protein levels of Vimentin and N-cadherin as well as inhibited the mRNA and protein levels of E-cadherin in HLFs, whereas these changes could be suppressed by PFD treatment (*P* < 0.001, Fig. 4a-c). Similar to the effect of TGF-β1, the addition of β-catenin reversed the function of PFD in EMT-related indices (*P* < 0.05, Fig. 4a-c).

### PFD protected HLFs from TGF-β1 damage via Wnt/GSK-3β/β-catenin and TGF-β1/Smad2/3 signaling pathways

In Wnt/GSK-3β/β-catenin and TGF-β1/Smad2/3 signaling pathways, WB analysis indicated that TGF-β1 promoted the mRNA and protein levels of p-GSK-3β S9, β-catenin, TGF-β1, TGF-βRΙ, TGF-βRΙΙ and p-Smad2/3 in HLFs, which could be rescued by the treatment of PFD (*P* < 0.01, Fig. 4d-j). On the contrary, β-catenin could eliminate the effect of PFD and enhance the protein levels of β-catenin, TGF-β1, TGF-βRΙ, TGF-βRΙΙ and p-Smad2/3 in cells (*P* < 0.05, Fig. 4d-j).

## Discussion

PFD, a small-molecule anti-fibrosis drug, has been proved to alleviate the degree of pulmonary fibrosis pathologically in the mouse fibrosis models induced by BLM (Inomata et al. 2014), but the specific mechanism of its anti-fibrosis effect is still not comprehensive, especially in the study of cell function. Current studies have shown that TGF-β1 is the main cytokine that promotes fibrosis with the strongest effect ever discovered (Meng et al. 2016). In related pathways, Smads family plays an important role in the extracellular matrix accumulation caused by TGF-β1 (Hata and Chen 2016). Furthermore, in previous findings, some scholars advocated that Wnt signaling pathway was abnormally activated in pulmonary fibrosis tissues, which was conducive to the production and release of TGF-β1 (Guan and Zhou 2017; Andersson-Sjoland et al. 2016). In Wnt signaling pathway family, GSK-3β S9 is an important member that belongs to glycogen synthase kinase, and its phosphorylation can impair the activity of GSK-3β and help activate β-catenin. As reported by Xiao H et al. (Xiao et al. 2018), PFD has been shown to effectively reduce the activation of pGSK-3β S9 and thus having an anti-fibrotic effect in patients with systemic sclerosis-associated interstitial lung disease (SSc-ILD). Therefore, it could be speculated that Wnt/GSK-3β/β-catenin and TGF-β1/Smad2/3 signaling pathways may be the crucial medium for PFD to play an anti-fibrosis role in PF.

To demonstrate our hypothesis, we first constructed mice models of pulmonary fibrosis using BLM, and found that the inflammatory response and extracellular matrix deposition of lung tissues in the mice models were extremely serious, which were consistent with the previous reports (Zhang et al. 2015a), indicating the success of in vivo models establishment. Interestingly, it was worth mentioning that the intervention of PFD not only significantly alleviated the inflammatory response and extracellular matrix deposition in the lung tissues of models, but also directly down-regulated the releases of inflammatory factors TNF-α and IL-6, whereas above conditions could be reversed by the addition of β-catenin. In addition, we also examined the levels of extracellular matrix deposition related indicators in lung tissues, and found that PFD correspondingly decreased the mRNA and protein levels of Col-I, Col-III, α-SMA and fibronectin in lung tissues induced by BLM. These results suggested that PFD had an obvious alleviation effect on PF, while up-regulation of β-catenin could lead to the aggravation of PF.

In Wnt pathway signaling, β-catenin can be phosphorylated and degraded by GSK-3β under normal conditions, which could be protected when GSK-3β S9 is phosphorylated, and thus gradually accumulating in the cytoplasm (Kim et al. 2017). This present study demonstrated that PFD inactivated BLM-induced GSK-3β/β-catenin pathway, suggesting that PFD could promote the degradation and reduce the accumulation of β-catenin through inhibiting the phosphorylation of GSK-3β S9, thereby relieving pulmonary fibrosis. As for the TGF-β1/Smad2/3 signaling pathway, TGF-β1 must bind to its receptor TGF-βR to form a transmembrane complex in order to exert fibrinogenic action, and there are three forms of TGF-βR in the body (I, II, III), among which TGF-βRI and TGF-βRII play an important role in cell signaling depending on Serine (Ser)/Threonine (Thr) protein kinases (Zhang et al. 2017; Akhmetshina et al. 2012). After the formation of TGF-β1-TGF-βRI-TGF-βRII trimer complex, its downstream Smad2/3 protein is phosphorylated, which means the excitation of TGF-β1/Smad2/3 signaling pathway, and then stimulates extracellular matrix deposition and visceral fibrosis (Biernacka et al. 2011). Here, we further illuminated that PFD had the effect of restraining the protein levels of TGF-β1, TGF-βRI, TGF-βRII, p-Smad2 and p-Smad3, which signifies that PFD blocked the activation of TGF-β1/Smad2/3 signaling pathway. Based on the above animal analysis, we could preliminarily know that PFD had a positive attenuating effect on BLM-induced pulmonary fibrosis, which was mainly achieved by inhibiting the phosphorylation of GSK-3β S9 and inactivating TGF-β1/Smad2/3 signaling pathway.

At the cellular level, it is currently believed that fibroblasts play an important role in the process of fibrosis, and their transformation into myofibroblasts can increase collagen synthesis and deposition in the alveolar septa, thereby causing pulmonary fibrosis (Pardo and Selman 2016). Therefore, HLFs in this study were exposed to TGF-β1 to induce cell phenotypic transformation, and then treated with PFD or β-catenin, so as to further explore the cellular and molecular mechanism of PFD treatment in pulmonary fibrosis. Consistent with animal experiments, on the one hand, PFD could reduce the activity of TGF-β1-induced HLFs; on the other hand, it can also decrease the extracellular matrix deposition-related proteins and mRNAs, including Col-I, Col-III, α-SMA and fibronectin. Moreover, this study also found that PFD inhibited the mRNA and protein levelss of vimentin and N-cadherin as well as promoted the mRNA and protein levels of E-cadherin while retarding the activation of Wnt/GSK-3β/β-catenin and TGF-β1/Smad2/3 signaling pathways. To the best of our knowledge, vimentin, N-cadherin and E-cadherin were indices related to EMT and often evaluated in fibrotic disease (Zhang et al. 2015b). Previous research results have confirmed that EMT was a key step in the progression of pulmonary fibrosis, and the levels of vimentin was elevated in IPF samples while E-cadherin level was depressed (Milara et al. 2012; Han et al. 2018). In related studies, Kurimoto R et al. (Kurimoto et al. 2017) reported that PFD could reduce the levels of E-cadherin and promote the levels of vimentin, thereby reversing the EMT of human lung adenocarcinoma and restoring the cell phenotype. Therefore, we hypothesized that PFD could regulate relevant signaling pathways and EMT in HLFs to regulate extracellular matrix deposition, thereby delaying the progression of pulmonary fibrosis.

There was also a limitation in this research, that was not setting a β-catenin group as positive control, as the degree of inflammatory cells infiltration seemed to be similar between model group and PFD + β-catenin group, which could be improved in future study.

## Conclusions

In summary, PFD not only alleviated pulmonary fibrosis of mice models caused by BLM, but also relieved TGF-β1-induced HLFs damage, which were mainly realized by regulating Wnt/GSK-3β/β-catenin and TGF-β1/Smad2/3 signaling pathways. This study elaborated the mechanisms and pathways involved in the treatment of pulmonary fibrosis with PFD, which might further improve the action mechanism of anti-fibrosis effect of PFD.

## Data Availability

The datasets used and/or analyzed during the current study are available from the corresponding author on reasonable request.

## Abbreviations

PFD: Pirfenidone
PF: Pulmonary fibrosis
BLM: Bleomycin
HLFs: Human fetal lung fibroblasts
EMT: Epithelial-mesenchymal transition
IPF: Idiopathic PF
HPS: Hermansky-pudlak syndrome
FVC: Forced vital capacity
TNF-α: Tumor necrosis factor-alpha
IL: Interleukin
HE: Hematoxylin-eosin
ELISA: Enzyme-linked immunosorbent assay
Col-I: Content of Collagen I
HRP: Horseradish peroxidase
DAB: Diaminobenzidine¬
NC: Negative control
MTT: Methylthiazolyldiphenyl-tetrazolium bromide
OD: Optical density
qRT-PCR: Quantitative real-time polymerase chain reaction
SDS-PAGE: Sodium dodecyl sulfate-polyacrylamide gel electrophoresis
PVDF: Polyvinylidene fluoride
SD: Standard deviation
ANOVA: Analysis of variance

## References

[CR1] Akhmetshina A, Palumbo K, Dees C, Bergmann C, Venalis P, Zerr P, Horn A, Kireva T, Beyer C, Zwerina J (2012). Activation of canonical Wnt signalling is required for TGF-beta-mediated fibrosis. Nat Commun.

[CR2] Andersson-Sjoland A, Karlsson JC, Rydell-Tormanen K (2016). ROS-induced endothelial stress contributes to pulmonary fibrosis through pericytes and Wnt signaling. Lab Invest.

[CR3] Biernacka A, Dobaczewski M, Frangogiannis NG (2011). TGF-beta signaling in fibrosis. Growth Factors (Chur, Switzerland).

[CR4] Gahl WA, Brantly M, Troendle J, Avila NA, Padua A, Montalvo C, Cardona H, Calis KA, Gochuico B (2002). Effect of pirfenidone on the pulmonary fibrosis of Hermansky-Pudlak syndrome. Mol Genet Metab.

[CR5] Guan S, Zhou J (2017). Frizzled-7 mediates TGF-beta-induced pulmonary fibrosis by transmitting non-canonical Wnt signaling. Exp Cell Res.

[CR6] Guo J, Yang Z, Jia Q, Bo C, Shao H, Zhang Z (2019). Pirfenidone inhibits epithelial-mesenchymal transition and pulmonary fibrosis in the rat silicosis model. Toxicol Lett.

[CR7] Han Q, Lin L, Zhao B, Wang N, Liu X (2018). Inhibition of mTOR ameliorates bleomycin-induced pulmonary fibrosis by regulating epithelial-mesenchymal transition. Biochem Biophys Res Commun.

[CR8] Hata Akiko, Chen Ye-Guang (2016). TGF-β Signaling from Receptors to Smads. Cold Spring Harbor Perspectives in Biology.

[CR9] Inomata M, Kamio K, Azuma A, Matsuda K, Kokuho N, Miura Y, Hayashi H, Nei T, Fujita K, Saito Y (2014). Pirfenidone inhibits fibrocyte accumulation in the lungs in bleomycin-induced murine pulmonary fibrosis. Respir Res.

[CR10] Jin J, Togo S, Kadoya K, Tulafu M, Namba Y, Iwai M, Watanabe J, Nagahama K, Okabe T, Hidayat M (2019). Pirfenidone attenuates lung fibrotic fibroblast responses to transforming growth factor-beta1. Respir Res.

[CR11] Kim ES, Keating GM (2015). Pirfenidone: a review of its use in idiopathic pulmonary fibrosis. Drugs.

[CR12] Kim JG, Kim MJ, Choi WJ, Moon MY, Kim HJ, Lee JY, Kim J, Kim SC, Kang SG, Seo GY (2017). Wnt3A induces GSK-3beta phosphorylation and beta-catenin accumulation through RhoA/ROCK. J Cell Physiol.

[CR13] Komiya C, Tanaka M, Tsuchiya K, Shimazu N, Mori K, Furuke S, Miyachi Y, Shiba K, Yamaguchi S, Ikeda K (2017). Antifibrotic effect of pirfenidone in a mouse model of human nonalcoholic steatohepatitis. Sci Rep.

[CR14] Kurimoto R, Ebata T, Iwasawa S, Ishiwata T, Tada Y, Tatsumi K, Takiguchi Y (2017). Pirfenidone may revert the epithelial-to-mesenchymal transition in human lung adenocarcinoma. Oncol Lett.

[CR15] Li C, Han R, Kang L, Wang J, Gao Y, Li Y, He J, Tian J (2017). Pirfenidone controls the feedback loop of the AT1R/p38 MAPK/renin-angiotensin system axis by regulating liver X receptor-alpha in myocardial infarction-induced cardiac fibrosis. Sci Rep.

[CR16] Li Z, Liu X, Wang B, Nie Y, Wen J, Wang Q, Gu C (2017). Pirfenidone suppresses MAPK signalling pathway to reverse epithelial-mesenchymal transition and renal fibrosis. Nephrology (Carlton, Vic).

[CR17] Lopez-de la Mora DA, Sanchez-Roque C, Montoya-Buelna M, Sanchez-Enriquez S, Lucano-Landeros S, Macias-Barragan J, Armendariz-Borunda J (2015). Role and new insights of Pirfenidone in fibrotic diseases. Int J Med Sci.

[CR18] Medina JL, Sebastian EA, Fourcaudot AB, Dorati R, Leung KP (2019). Pirfenidone ointment modulates the burn wound bed in C57BL/6 mice by suppressing inflammatory responses. Inflammation.

[CR19] Meier R, Lutz C, Cosin-Roger J, Fagagnini S, Bollmann G, Hunerwadel A, Mamie C, Lang S, Tchouboukov A, Weber FE (2016). Decreased Fibrogenesis after treatment with Pirfenidone in a newly developed mouse model of intestinal fibrosis. Inflamm Bowel Dis.

[CR20] Meng XM, Nikolic-Paterson DJ, Lan HY (2016). TGF-beta: the master regulator of fibrosis. Nat Rev Nephrol.

[CR21] Meyer KC (2017). Pulmonary fibrosis, part I: epidemiology, pathogenesis, and diagnosis. Expert Rev Respir Med.

[CR22] Milara J, Navarro R, Juan G, Peiro T, Serrano A, Ramon M, Morcillo E, Cortijo J (2012). Sphingosine-1-phosphate is increased in patients with idiopathic pulmonary fibrosis and mediates epithelial to mesenchymal transition. Thorax.

[CR23] Noble PW, Barkauskas CE, Jiang D (2012). Pulmonary fibrosis: patterns and perpetrators. J Clin Invest.

[CR24] Pardo A, Selman M (2016). Lung fibroblasts, aging, and idiopathic pulmonary fibrosis. Ann Am Thorac Soc.

[CR25] Rao X, Lai D, Huang X (2013). A new method for quantitative real-time polymerase chain reaction data analysis. J Comput Biol.

[CR26] Richeldi L, Collard HR, Jones MG (2017). Idiopathic pulmonary fibrosis. Lancet (London, England).

[CR27] Stahnke T, Kowtharapu BS, Stachs O, Schmitz KP, Wurm J, Wree A, Guthoff RF, Hovakimyan M (2017). Suppression of TGF-beta pathway by pirfenidone decreases extracellular matrix deposition in ocular fibroblasts in vitro. PLoS One.

[CR28] Tomos IP, Tzouvelekis A, Aidinis V, Manali ED, Bouros E, Bouros D, Papiris SA (2017). Extracellular matrix remodeling in idiopathic pulmonary fibrosis. It is the ‘bed’ that counts and not ‘the sleepers’. Expert Rev Respir Med.

[CR29] Xiao H, Zhang GF, Liao XP, Li XJ, Zhang J, Lin H, Chen Z, Zhang X (2018). Anti-fibrotic effects of pirfenidone by interference with the hedgehog signalling pathway in patients with systemic sclerosis-associated interstitial lung disease. Int J Rheum Dis.

[CR30] Zhang L, Ji YX, Jiang WL, Lv CJ (2015). Protective roles of pulmonary rehabilitation mixture in experimental pulmonary fibrosis in vitro and in vivo. Braz J Med Biol Res.

[CR31] Zhang Q, Ye H, Xiang F, Song LJ, Zhou LL, Cai PC, Zhang JC, Yu F, Shi HZ, Su Y (2017). miR-18a-5p inhibits sub-pleural pulmonary fibrosis by targeting TGF-beta receptor II. Mol Ther.

[CR32] Zhang Z, Yu X, Fang X, Liang A, Yu Z, Gu P, Zeng Y, He J, Zhu H, Li S (2015). Preventive effects of vitamin D treatment on bleomycin-induced pulmonary fibrosis. Sci Rep.

